# The Effect of Meclofenoxate on the Transcriptome of Aging Brain of *Nothobranchius guentheri* Annual Killifish

**DOI:** 10.3390/ijms23052491

**Published:** 2022-02-24

**Authors:** Ildar R. Bakhtogarimov, Anna V. Kudryavtseva, George S. Krasnov, Natalya S. Gladysh, Vsevolod V. Volodin, Alexander A. Kudryavtsev, Elizaveta V. Bulavkina, Margarita A. Goncharova, Veronika S. Ledyaeva, Ivan S. Pastukhov, Yulia S. Vershinina, Anna M. Starkova, Anastasiya V. Snezhkina, Anastasija I. Shuvalova, Vladislav S. Pavlov, Dmitry L. Nikiforov-Nikishin, Alexey A. Moskalev, Zulfiya G. Guvatova

**Affiliations:** 1 Center for Precision Genome Editing and Genetic Technologies for Biomedicine, Engelhardt Institute of Molecular Biology, Russian Academy of Sciences, 119991 Moscow, Russia; bakhtogarimov@gmail.com (I.R.B.); gskrasnov@mail.ru (G.S.K.); natalyagladish@gmail.com (N.S.G.); vsevolodvolodin@yandex.ru (V.V.V.); amoskalev@list.ru (A.A.M.); 2 Engelhardt Institute of Molecular Biology, Russian Academy of Sciences, 119991 Moscow, Russia; aleks.kudrjavcev@gmail.com (A.A.K.); bulavkinaelizaveta8@gmail.com (E.V.B.); make.science.not.stereotypes@gmail.com (M.A.G.); veronikaledya@mail.ru (V.S.L.); yulia_vershinina@list.ru (Y.S.V.); staranna2001@mail.ru (A.M.S.); leftger@rambler.ru (A.V.S.); anastasiashuvalova777@gmail.com (A.I.S.); vladislav1pavlov@gmail.com (V.S.P.); 3 Institute of Biotechnology and Fisheries, K.G. Razumovsky Moscow State University of Technologies and Management (the First Cossack University), 109004 Moscow, Russia; ivanpeskar@gmail.com (I.S.P.); 9150699@mail.ru (D.L.N.-N.)

**Keywords:** *Nothobranchius guentheri*, brain aging, meclofenoxate, RNA-Seq

## Abstract

Annual fish of the genus *Nothobranchius* are promising models for aging research. *Nothobranchius* reproduces typical aspects of vertebrate aging, including hallmarks of brain aging. Meclofenoxate (MF) is a well-known compound that can enhance cognitive performance. The drug is prescribed for asthenic conditions, trauma, and vascular diseases of the brain. It is believed that MF is able to delay age-dependent changes in the human brain. However, until now, there has been no study of the MF effect on the brain transcriptome. In the present work, we performed an RNA-Seq study of brain tissues from aged *Nothobranchius guentheri*, which were almost lifetime administered with MF, as well as young and aged control fish. As expected, in response to MF, we revealed significant overexpression of neuron-specific genes including genes involved in synaptic activity and plasticity, neurotransmitter secretion, and neuron projection. The effect was more pronounced in female fish. In this aspect, MF alleviated age-dependent decreased expression of genes involved in neuronal activity. In both treated and untreated animals, we observed strong aging-associated overexpression of immune and inflammatory response genes. MF treatment did not prevent this effect, and moreover, some of these genes tended to be slightly upregulated under MF treatment. Additionally, we noticed upregulation of some genes associated with aging and cellular senescence, including isoforms of putative vascular cell adhesion molecule 1 (VCAM1), protein O-GlcNAcase (OGA), protein kinase C alpha type (KPCA), prolow-density lipoprotein receptor-related protein 1 (LRP1). Noteworthy, MF treatment was also associated with the elevated transcription of transposons, which are highly abundant in the *N. guentheri* genome. In conclusion, MF compensates for the age-dependent downregulation of neuronal activity genes, but its effect on aging brain transcriptome still cannot be considered unambiguously positive.

## 1. Introduction

Fish of the genus *Nothobranchius*, so-called killifish, are the fastest maturing vertebrates and have a short lifespan, which is obviously associated with adaptation to the seasonal rains in habitats. Numerous behavioral, histochemical, and genetic studies have revealed that *Nothobranchius* exhibit signs of age-related deterioration, including loss of body mass, reproductive dysfunction, a high incidence of tumors with age, as well as cognitive degeneration [1]. In addition, a useful feature of fish is sensitivity to temperature variations, which makes it relatively easy to control their aging rate in a laboratory setting [2,3].

*Nothobranchius guentheri* is one of the long-standing fish models for aging research with an average lifespan of 12 month. As early as 1970s, *Markofsky* et al. described age-related histological changes in a wide range of *N. guentheri* organs such as the eyes, liver, kidneys, and thymus [4,5,6]. Subsequent studies have shown that *N. guentheri* demonstrated changes in the expression of aging biomarkers [7,8,9] making this species suitable for testing anti-aging interventions [10,11] and for studying various aspects of aging, including brain aging [12,13]. On the one hand, it is known that the brain of teleost fish, in contrast to mammals, shows widespread adult neurogenesis and neural regeneration [14]. On the other hand, studies on *Nothobranchius furzeri*, the shortest-lived species of *Nothobranchius*, have shown several signs of brain aging including reduced learning performances, age-dependent gliosis, and reduced adult neurogenesis [15]. RNA-Seq studies of *N. furzeri* have revealed aging-associated changes in pathways and genes associated with neurogenesis [16,17]. As for other *Nothobranchius* species, there are still no studies of gene expression alteration during brain aging.

In this work, we studied the effect of prolonged oral administration of meclofenoxate (MF, also known as centrophenoxine) on the transcriptome of *N. guentheri* aging brain. MF as a cholinergic nootropic drug has been in clinical use for many years [18]. MF has been shown to improve impaired cognitive performance, including attention, concentration, memory, and performance IQ [19]. Clinically, MF is used for asthenic conditions, traumatic and vascular diseases of the brain, diencephalic syndrome, amyotrophic lateral syndrome, obsessive neuroses, and other neurotic disorders. The peak of research on MF occurred during 1960—1990. MF has also demonstrated anti-aging properties, such it was shown on pig and mouse models [20]. MF has been shown to decrease lipofuscin formation, the process that is one of the hallmarks of aging, and its metabolites can exhibit antioxidant activity [19,21].

However, until now, there were no transcriptomic studies on the MF effects. In this work, to elucidate the molecular aspects of MF treatment on the brain of *N. guentheri*, we compared brain transcriptomes of aged fish fed a lifelong diet containing MF to the brain transcriptomes of control group fish. Then, we juxtaposed the derived results with the observed age-associated gene expression changes for the control group.

## 2. Results and Discussion

### 2.1. De Novo Transcriptome Assembly and Annotation

In the present work, we performed three transcriptome assemblies using several RNA-Seq datasets derived by us for *N. guentheri*: (a) RNA-Seq data from the current work (29 Gbases), (b) RNA-Seq data from a previous study on the effect of mTOR inhibitor torin2 on adult fish brain transcriptome (38 Gbases) [22], and (c) a combination of these two datasets with additional *N. guentheri* RNA-Seq datasets (unpublished, total 48 Gbases). All RNA-Seq data include single-end reads (75 nt).

Assembly statistics (QUAST) and quality control metrics (rnaQUAST, BUSCO) are presented in Table 1. The overall number of assembled transcripts was almost proportional to the dataset size. For the current dataset, we derived 226,000 transcripts; for the dataset from the torin2 study, we derived 352,000 transcripts; and for the joint pool, we derived 595,000 transcripts. At the same time, for long transcripts, this ratio softens, and for very long ones (>5, 10 Kbases) it even turns out in favor of the dataset from the torin2 study. Unsurprisingly, we obtained the worst N50 and L50 values for the joint dataset.

Thus, the assembly based on the joint pool of data is littered with many short transcripts, which in most cases are weakly expressed. One cannot ignore the fact that the *N. guentheri* genome is very rich in transposons, and the transcribed intron and intergenic regions related to transposons may be the source of these transcripts. In addition, another reason may be the fact that the joint dataset includes RNA-Seq reads derived from several dozen individual organisms (and also includes non-brain tissues). This may be associated with the presence of genetic variability and the complexity of alternative splicing patterns.

Considering rnaQUAST metrics, the differences in assembly quality were not so significant. Thus, for all three assemblies, 99.3—99.8% of the transcripts were successfully aligned by GMAP to the reference genome of *N. guentheri* female. However, the number of misassembles per 1 Mb varied from 37 to 53 and was almost proportional to the dataset size. Thus, taking into consideration all of the above, we can say that starting from a certain size of the dataset used for assembly, the assembly quality may even deteriorate. Ideally, one should choose the optimal dataset size to get the best assembly quality. However, in our case, for differential expression analysis, it is mostly important to include the maximum number of genes. Therefore, the completeness of the assembly assessed by BUSCO is crucial.

As expected, the best BUSCO results were observed for *Eukaryota* and *Metazoa* gene sets (about 97% completed BUSCOs). They contain conservative single copy orthologs, which have less tissue specificity of expression. For the most detailed “fish-specific” gene sets *Actinopterygii* and *Cyprinodontiformes*, the best result was noted for the joint dataset, with differences up to 7% between the assemblies. A possible reason for this is the presence of RNA-Seq reads derived from non-brain tissues (in the joint dataset). For the assembly performed using the dataset from this work, the worst completeness according to BUSCO was noted, which was an indicator of too little data used for assembly (29 Gb). In general, the obtained results for the joint dataset suggest enough coverage of transcripts for the subsequent analysis of differential expression. This is also evidenced by ExN50 statistics (data not shown).

**Figure 1:**
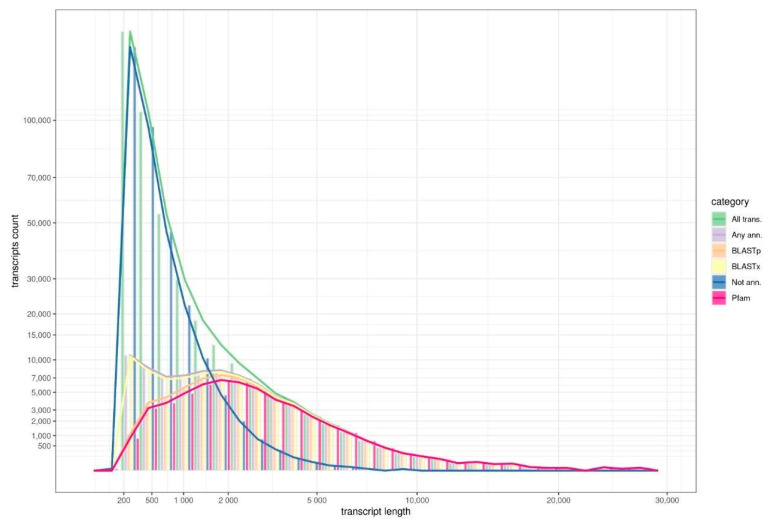
The histogram illustrates the distribution of the assembled transcripts depending on their length (bp) and availability of annotation using UniProt (BLASTp, BLASTx) or Pfam (HMMER) databases.

Next, we annotated the assembled transcripts using the Trinotate pipeline. As expected, the rate of annotation was significantly higher for long transcripts (Figure 1). Thus, almost all transcripts longer than 3000 bp were successfully annotated with all three algorithms (BLASTx and BLASTp versus UniProt; HMMER versus Pfam). For short transcripts (less than 500 nuclei), BLASTx provided significantly more annotations than the other two methods (Figure 1). However, the reliability of such annotations is lower than for others. In general, the percentage of annotated short transcripts (200–500 bp) remains low (less than 10%).

### 2.2. Age-Dependent Gene Expression Changes in N. guentheri Brain in Meclofenoxate-Treated Fish and Controls

In the control group, we compared the gene expression profiles of *N. guentheri* brain at two time points, i.e., 3 and 12 months. Females and males were analyzed separately, since the gene expression profile during aging can differ significantly between the sexes [23]. Indeed, females showed stronger age-dependent transcriptomic changes (2200 genes with FDR < 0.05, including 1455 genes with at least 1.5-fold change) than males, for which only 1180 and 768 differentially expressed genes (DEG) were found, respectively.

Next, in aged fish (12 months), we compared gene expression profiles between MF-treated fish and the control group. Here, we also noticed a greater difference for females. Thus, for females, 783 DEGs with FDR < 0.05 (524 of them have at least 1.5-fold change) were identified, while for males, more modest results were obtained, i.e., only 128 (126) DEGs were noted. Partly, such differences are due to the fact that the size of the MF-treated group for females was larger (four animals) than for males (three animals), since *p*-values depend on a sample size. Sexual dimorphism is a common phenomenon exhibited in diverse characteristics, including the structure and function of the central nervous system (CNS) [23]. It is assumed that different brain tissues have different aging rates between sexes, which is also reflected in differences in the gene expression profiles [24]. Moreover, many CNS disorders show sex differences in their incidence or nature [25]. It is not surprising that we found significant sex differences in the brain transcriptomes of fish treated using the nootropic drug. For females, a predominant increase in expression was noted (on average, a two-fold increase for all DEGs that passed the FDR < 0.05 threshold). Thus, 642 of 783 DEGs (82%) were upregulated as a result of the MF diet. For males, only 85 of 128 DEGs (74%) demonstrated increased expression. It is noteworthy that in the case of cross-comparison MF-treated aged fish vs. non-treated young animals, we derived a lower number of DEGs than in the case of non-treated aged versus young organisms, which could indicate a softening of age-related changes by MF.

The gene set enrichment analysis (GSEA) revealed several cell pathways and biological processes most likely altered during aging. Pathways related to circadian rhythm, immune and inflammatory response, antigen processing and presentation, E-box binding, and translation, were highly enriched with genes upregulated during aging. Dozens of pathways related to histone methylation and TCA cycle were enriched with genes whose expression decreases with age. The GSEA analysis revealed that the downregulated DEGs (FDR < 0.05) were mostly enriched in terms such as the regulation of neurogenesis (GO:0050767), axonogenesis (GO:0050770), neuron differentiation (GO:0045664), and synapse organization (GO:0050807), which may indicate an age-related decrease in adult neurogenesis in *N. guentheri*. Indeed, it has been previously shown that, in fish of the genus *Notobranchius*, at least in *N. furzeri*, there is a decline in neurogenic activity with aging [16,26], while other popular fish model zebrafish show extensive adult neurogenesis and neuronal regeneration [14]. Among downregulated DEGs with age, we also detected the genes *NEFL, NEFH,* and *NEFM*, encoding neurofilament subunits. Completely eliminating neurofilament proteins from the brain disrupted synaptic plasticity and impaired social memory in mice [27,28]. In another study, it was shown that a decline in the expression of these genes was associated with a decrease in the number of myelinated axons [29], which was crucial to adult neuron performance. Moreover, tightening of the LogFC threshold revealed many other genes encoding cytoskeletal proteins. According to the results, the expression of genes (|LogFC| ≥ 1, FDR < 0.05) related to microtubule-associated protein (MABP1) and actin (ACTS, ACTN1, ACTN3, and ACTB); keratin (K1C1 and K1C13); and myosin (MYSS, MLE3, and MLRS) proteins was decreased with age more than two times. The impact of actin dynamics on the life span has been reported in several studies on yeast, mice, and rats [30]. As regards the nervous system, cytoskeletal function and dynamics are essential for the morphological and functional plasticity of neurons and glial cells [31]. It is no wonder that dysfunction of the cytoskeleton is associated with age-associated changes in the brain and implicated in diverse neurodegenerative diseases [32,33]. 

**Figure 2:**
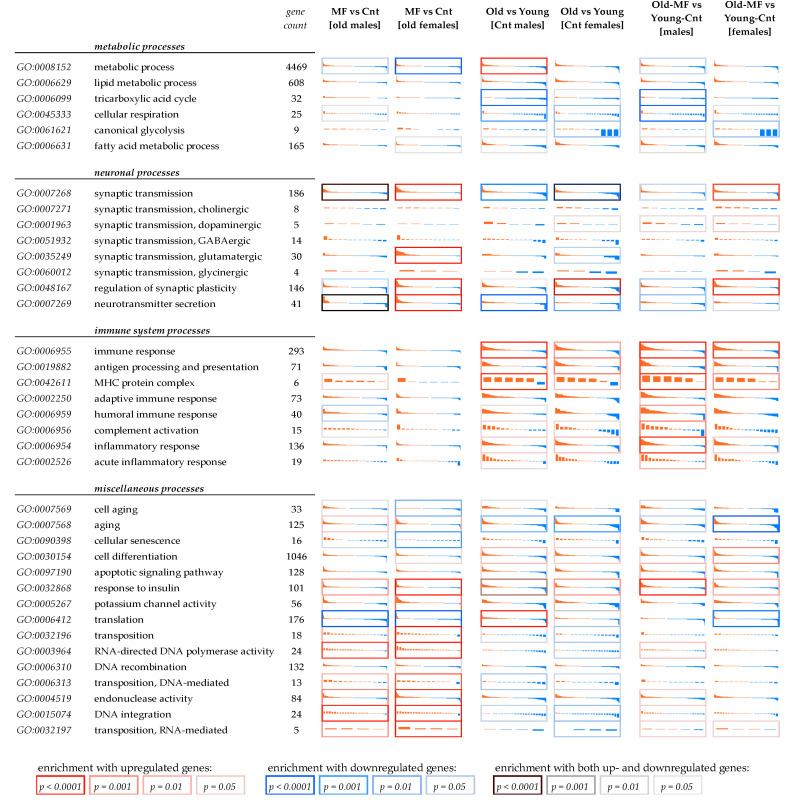
Differential expression profiles of genes involved in various biological processes mostly affected either during aging or meclofenoxate treatment. Each cell demonstrates differential expression profiles for genes involved in a current GO biological process, either upregulated (red) or downregulated (blue) in (MF) fish with lifetime meclofenoxate-containing food supplement as compared with control (Cnt) fish, or in 12-month fish as compared with 3-month fish. Relative expression values are log2-transformed. Vertical axis range (in each cell) is from −2 (i.e., 4-fold expression increase) to +2 (i.e., 4-fold expression decrease). Genes are sorted according to LogFC. Cell borders indicate enrichment test results significance.

Figure 2 demonstrates GO-centric differential expression profiles for biological processes mostly enriched either with DEGs after MF treatment or aging-associated DEGs.

Unsurprisingly, as can be seen from the Figure 2, MF supplement results in the increased expression of genes involved in synaptic transmission and neurotransmitter secretion. It is known that MF stimulates glucose uptake, oxygen consumption, and enhances brain energy metabolism [34,35]. Hence, it exerts an overall neuronal activity stimulating effect. Dimethylaminoethanol, the active component of meclofenoxate, is a close structural analog of choline, precursor for acetylcholine. Therefore, first of all, one should expect upregulation of genes involved in cholinergic neuronal transmission. However, we did not observe differential expression of either cholinergic or dopaminergic synaptic transmission genes. In contrast, in female organisms, we noticed strong upregulation (from two to four fold) of many genes involved in glutamatergic synaptic transmission: subunits of ionotropic glutamate receptor genes, such as *GRIN1, GRIN2D,* (NMDA receptors), and *GRIK3* (kainite receptors); voltage-dependent calcium channels genes *CAC1A* and *CAC1B*. Regarding genes involved in cholinergic transmission, we noticed only small (less than 1.3-fold) and not significant (FDR > 0.05) upregulation of acetylcholine receptor subunit alpha-7 (*ACHA7*) and high affinity choline transporter 1 (*SC5A7*).

Glutamate is very abundant in a human organism and it is the most prominent neurotransmitter in the human nervous system. Ionotropic glutamate receptors represent ligand-dependent ion channels and are important for synaptic plasticity, memory, and learning [36]. They mediate most excitatory synaptic transmission throughout the CNS. Hence, this mechanism may stand behind the positive effect of MF on cognitive function and memory.

Among the biological processes that are highly enriched with genes upregulated during aging, the immune and inflammatory response, and especially genes forming the MHC complex take first place. It is well known that aging is characterized by unresolved and uncontrolled chronic inflammation [37], a shift from the homeostatic balance of inflammatory mediators to a proinflammatory state [38].

Neuroinflammation, an inflammation of the nervous tissue, usually results from infections, trauma, toxins, and autoimmune diseases [39,40]. It is a common feature of neurodegenerative disorders, including Alzheimer’s, Parkinson’s, and Huntington diseases [41]. Neuroinflammation is tightly related with aging, it makes CNS prone to the effects of stress and infections [38]. Microglia play a pivotal role in neuroinflammation associated with the development of dementia. Activation of microglia has been shown to initiate a primary inflammatory response and cause secondary leukocyte invasion, which increased inflammation [37,42]. MF supplement did not result in any attenuation in the age-dependent overexpression of immune and inflammatory response genes, which indicated the possible absence of MF influence on these processes that are often activated during aging. Moreover, under MF treatment, there was a slight trend towards an increase in the expression of genes for the humoral immune response and a more pronounced trend towards an increase in the expression of genes encoding subunits of the MHC complex (Figure 2). Noteworthily, among immune-related pathways, we noticed the most striking age-associated overexpression just for the subunits of MHC. It is known that upregulation of MHC-II occurs during normal brain aging in CNS resident macrophages, at both mRNA and protein levels, and indicates a shift towards a proinflammatory microenvironment in the CNS [43,44], whereas in young organisms, MHC-II is expressed in microglia at very low levels. This has been shown for human and various animal models (primates, canine, and rodents) [43,45]. 

As seen from the Figure 2, meclofenoxate treatment resulted in the increased expression of several genes associated with aging and cellular senescence, including isoforms of protein O-GlcNAcase (OGA), protein kinase C alpha type (KPCA), prolow-density lipoprotein receptor-related protein 1 (LRP1), and vascular cell adhesion molecule 1 (VCAM1). Some of these genes play pivotal roles in neurodegenerative disorders. First of all, we are talking about *VCAM1*, a key immune system player that is expressed in endothelial cells in response to cytokines stimulus, for example IL-1 and TNF [46]. It mediates adhesion of leukocytes including lymphocytes and monocytes. VCAM1 is implicated in the inflammatory response, development and progression of various immunological disorders, including rheumatoid arthritis, asthma, and neurodegenerative diseases [47,48]. Recently it was shown that the expression of VCAM1 on brain endothelial cells increased with age, whereas inactivation of VCAM1 attenuated microglia activation and restored cognitive function in aged mice [49].

Next, we noticed that MF treatment was associated with an elevated expression of genes participating in DNA transposition. In particular, we found upregulated DNA polymerase from transposons BS and X-element, transposase from transposons Tc1 and Tc3, polyprotein from transposon Tf2–9, and many others. It is known that the genome of *Nothobranchius furzeri*, a close relative of *N. guentheri* is highly enriched with tandem repeats that comprise 21% of the genome length, which is 4–12 times greater than for other fish species such as zebrafish, medaka, stickleback, and tetraodon [50]. Known transposons occupy about 8% of the *N. furzeri* genome length. There are no unambiguous associations between transposon activity and aging for any organism, but many studies have indicated that aging was indeed associated with the loss of repressive structures in constitutive heterochromatin regions, and the following activation of transposable elements [51]. Age-specific reductions in lamins A, B, and C, which are necessary for maintaining heterochromatin, underly the decline in the suppression of transposon activity [52]. According to the results of the present study, we noticed MF-induced upregulation of several histone-lysine N-methyltransferases, positive global regulators of gene transcription. In theory, these changes could help compensate for age-specific gene expression downregulation, but they can also provoke the activation of the expression of various genes and genomic regions, including transposons.

In summarize, this study is the first report on age-dependent gene expression alterations of *N. guentheri* brain, as well the effect of meclofenoxate on brain transcriptome. In general, the obtained data are consistent with previously published data on conserved downregulation of synapse, mitochondrion, and cytoskeletal gene expression with age [30,48]. We also detected age-dependent changes in expression of genes related to circadian rhythms, immune and inflammatory response, DNA binding, and protein metabolic process. Meclofenoxate exerted a variety of transcriptomic effects, only some of which can be regarded as positive in the context of aging.

## 3. Materials and Methods

### 3.1. Fish Diet and Maintenance

In this work, we used the Zanzibar TAN 14–02 strain of *N. guentheri*. All fish were bred and kept under identical conditions. The fish were kept at 27 °C under a 14:10 h light/dark regime in a commercial aquatic housing system (Aquaneering, Inc., San Diego, CA, USA). The feeding of fish larvae was carried out 3 times per day with newly hatched brine shrimp *Artemia salina*. At the age of 1 month old, when fish attained sexual maturation, 30 control fish (15 females and 15 males) were switched to a diet with shredded agarose gel pieces containing nauplii of *A. salina* in the morning and red mosquito larvae (*Chironomidae*) in the evening. At the same time 30 experimental fish were switched to a diet containing MF at 100 mg/kg in 2/2-week cycles of treatment. During periods of treatment, we fed the fish a minimum amount of food so that they could eat all gel pieces containing MF. The experimental diet was prepared as follows: An agarose gel (0.8%) containing nauplii and MF was passed through a sieve with a mesh of approximately 1 mm2 for grinding. The *N. guentheri* experiments were all carried out in accordance with the recommendations described in the Guide for the Care and Use of Laboratory Animals [53] and were approved by the Ethics Committee of the A.N. Severtsov Institute of Ecology and Evolution Russian Academy of Sciences (Experimental Research Regulatory Comission of Institute of Ecology and Evolution A.N. Severtsov, approval number 27, 9 October 2019).

### 3.2. RNA Isolation, Library Preparation, and Transcriptome Sequencing 

For the transcriptomic analysis, two age groups of *N.guentheri* were used: 3 months old (4 males and 3 females), and 12 months old (4 males and 4 females). To access the effect of meclofenoxate, we also used 3 males and 4 females of aged *N. guentheri*, kept on an experimental diet. Brains were dissected and stored at −80 °C until the RNA isolation. To avoid influencing the results of diurnal fluctuations in gene expression, all fish were dissected at the same time of day.

Total RNA was isolated from the brain tissue samples using a MagNA Pure Compact RNA Isolation Kit (Roche, Basel, Switzerland), according to the manufacturer’s protocol. The quantity and quality of RNA were evaluated using a Qubit^®^2.0 Fluorometer (Thermo Fisher Scientific, Waltham, MA, USA) and Agilent 2100 Bioanalyzer (Agilent Technologies, Santa Clara, CA, USA). The RNA integrity number (RIN) of each sample was >8.0.

Double stranded cDNA libraries were prepared using an Illumina TruSeq Stranded Total RNA Library Prep Kit (Illumina, San Diego, CA, USA), according to the manufacturer’s guidelines from 0.3 μg of total RNA. The concentration of the 31 obtained cDNA libraries was assessed using a Qubit^®^2.0 Fluorometer (Thermo Fisher Scientific, Waltham, MA, USA). The quality of libraries was defined using a High Sensitivity DNA chip on an Agilent 2100 Bioanalyzer (Agilent Technologies, Santa Clara, CA, USA), according to the manufacturer’s protocol. Then, cDNA libraries were normalized to 4 nM, pooled together in equal volumes, and sequenced with 75-bp single-end reads on an NextSeq 500 System (Illumina, San Diego, CA, USA). The sequencing data derived in the present work are available at the NCBI Sequence Read Archive (project ID PRJNA779252).

### 3.3. NGS Data Processing

The transcriptome assembly was carried out with Trinity 2.9.0 [54] with switched off reads normalization. In order to enhance the completeness of the transcriptome assembly, we included the assembly not only Illumina reads derived from 21 RNA-Seq libraries from the present work (total 29 Gbases after trimming and filtering), but also RNA-Seq data from our previous *N. guentheri* work [22] (38 Gb), and several other RNA-Seq libraries from unpublished studies (total 48 Gb), derived from brain and non-brain tissues.

For the assembled transcripts, the longest ORFs were predicted using TransDecoder 5.5.0. The annotation of transcripts and ORFs was performed using Trinotate pipeline based on blastx/blastp mappings against UniProt and HMMER homology search against Pfam databases. From these sources, possible gene names, KEGG, and GO annotation were fetched. The completeness of the transcriptome assembly was assessed with BUSCO 4.0.6 (in transcriptome mode) using five datasets: *Eukaryota*, *Metazoa*, *Vertebrata*, *Actinopterygii*, and *Cyprinodontiformes* (odb10). Additionally, we calculated assembly metrics with QUAST and rnaQUAST. We used the earlier assembled female *N. guentheri* genome (unpublished, based on Nanopore reads) as a reference to assess the assembly quality with rnaQUAST.

To evaluate gene expression profiles, Illumina reads were mapped to the assembled transcripts using bowtie2 [55], and then quantified with RSEM [56]. Age-dependent and meclofenoxate-associated gene expression profile changes were identified with the ”edgeR” Bioconductor package [57], separately for males and females. A quasi-likelihood F-test (edgeR’s default) was done to assess the statistical significance of the observed expression changes, and then the Benjamini–Hochberg adjustment was applied to the derived *p*-values in order to calculate FDR. The GO enrichment analyses were carried out using the ”goseq” Bioconductor package.

We noticed that, in many cases, multiple assembled transcripts were joined into several “genes” that had the same name (derived from the best UniProt or Pfam hit) and often had similar expression patterns. Among them, there are genes that are expected to be single copy, which clearly indicates an assembly bias. The transcripts related to these eponymous “genes” are most likely derived from one “real gene”. In the enrichment analyses, these cases would be considered to be several separate entries, each of which has a common BLAST hit and inherit the same GO and KEGG annotations. Hence, this exerts a strong bias on the enrichment analysis and leads to false positive results. In order to eliminate this bias, we merged gene expression data (summed CPM values) for such “genes” with common predicted “real” names (e.g., ACTB and SDHD) derived from UniProt or Pfam mapping.

## Figures and Tables

**Table 1:** Assembly statistics of *Nothobranchius guentheri* transcriptome according to QUAST, rnaQUAST, and BUSCO metrics.

QUAST Metrics	Joint Dataset, 115 Gb	Current Dataset, 29 Gb	Dataset of the Work [22], 38 Gb
Transcripts (>0 bp)	595,883	226,597	352,297
Transcripts (>500 bp)	184,805	89,658	127,262
Transcripts (>1000 bp)	81,223	50,507	66,471
Transcripts (>5000 bp)	5757	3769	6322
Transcripts (>10,000 bp)	500	299	604
Transcripts (>25,000 bp)	10	6	5
Total length (>0 bp)	386,870,887	195,140,104	285,906,387
Total length (>500 bp)	263,650,013	153,792,134	217,410,128
Total length (>1000 bp)	192,720,831	126,643,405	175,623,569
Total length (>5000 bp)	40,282,776	26,395,594	45,099,163
Total length (>10,000 bp)	6,597,510	4,117,143	7,821,995
Total length (>25,000 bp)	268,609	162,941	134,557
Largest transcript	27,943	27,845	27,376
N50	1927	2423	2539
N75	948	1283	1231
L50	36,670	18,767	24,581
L75	86,380	40,530	55,134
% Length (>500 bp)	68.15	78.81	76.04
% Length (>1000 bp)	49.82	64.90	61.43
% Length (>5000 bp)	10.41	13.53	15.77
% Length (>10,000 bp)	1.71	2.11	2.74
% Length (>25,000 bp)	0.07	0.08	0.05
**rnaQUAST metrics**			
Aligned, %	99.29	99.63	99.78
Uniquely aligned, %	93.74	94.16	95.04
Multiply aligned, %	2.05	2.23	1.03
Unaligned, %	0.71	0.37	0.22
Avg. aligned fraction	0.9860	0.9880	0.9850
Avg. alignment length	615.47	818.24	769.95
Avg. mismatches per transcript	2.90	1.99	3.00
Misassemblies	20 848	7 336	13 051
Misassemblies per 1 Mb	53.89	37.59	45.65
**Completed BUSCOs, %**			
*Cyprinodontiformes*	66.96	59.19	63.94
*Actinopterygii*	88.38	82.83	86.68
*Vertebrata*	90.58	87.75	90.88
*Metazoa*	97.17	97.27	97.69
*Eukaryota*	96.86	96.08	98.04
**BUSCO duplication ratio, %**			
*Cyprinodontiformes*	33.49	29.16	36.52
*Actinopterygii*	32.58	30.38	37.75
*Vertebrata*	33.25	32.18	39.37
*Metazoa*	28.26	26.40	32.62
*Eukaryota*	25.10	32.65	33.60
cell colors	worse	middle	better

## Data Availability

The sequencing data are available at the NCBI Sequence Read Archive (project ID PRJNA779252).
